# Forecasting hospital bed occupancy: a time series approach with prophet

**DOI:** 10.1186/s12911-026-03542-w

**Published:** 2026-05-08

**Authors:** Mohammad Fattouh, L. Lyssenko, F. Heilmeyer, T. Ball, Ch. Haverkamp

**Affiliations:** 1 Institute of Digitalization in Medicine, Faculty of Medicine and Medical Center, University of Freiburg, Freiburg, Germany Breisacher Str. 64, 79106; 2 Neuromedical A.I. Lab, Department of Neurosurgery, Medical Center—University of Freiburg, Faculty of Medicine, University of Freiburg, Freiburg, Germany; 3 BrainLinks-BrainTools, IMBIT (Institute for Machine-Brain Interfacing Technology), University of Freiburg, Freiburg im Breisgau, Germany

**Keywords:** Hospital bed occupancy, Time series forecasting, Prophet, Operationalization, Hospital management

## Abstract

**Background:**

Accurate hospital bed occupancy forecasting is essential for effective resource planning and patient flow management. While complex machine learning models have gained popularity in healthcare forecasting, their operational utility often falls short due to high maintenance costs and limited interpretability. This study evaluates the performance and practicality of Prophet, a parsimonious time-series model, for mid-term hospital bed occupancy forecasting.

**Methods:**

We applied the Prophet model to daily bed occupancy data from the Medical Center – University of Freiburg (2010–2023), incorporating public holidays and a COVID-19 pandemic indicator as exogenous regressors. Prophet decomposes time series into trend, seasonality, and holiday effects, offering interpretable components. Forecast accuracy was assessed via rolling cross-validation over 2022–2023 for horizons of 30, 60, 90, and 180 days. A production-ready forecasting pipeline and dashboard were also implemented using cloud-native tools.

**Results:**

Prophet achieved low MAPE values across all horizons (3.21%–3.53%) with coverage above 80%, demonstrating reliable accuracy comparable to or better than more complex models that often require higher computational resources and operational costs, such as deep neural networks. Component analysis revealed patterns aligned with hospital operations; weekly and yearly cycles, and holiday effects, highlighting the model’s interpretability.

**Conclusions:**

This study shows that mid-term hospital bed occupancy can be accurately forecasted using a simple, interpretable model like Prophet. In contrast to more complex architectures, Prophet offers robust performance with minimal tuning, faster deployment, and clearer insights that are critical in operational settings. These findings reinforce the argument that, for structured forecasting tasks like bed occupancy, simple models can rival complex ones, not only in accuracy, but also in reproducibility, scalability, and operational value.

**Supplementary Information:**

The online version contains supplementary material available at 10.1186/s12911-026-03542-w.

## Background

The efficient allocation of healthcare resources represents a central challenge for health systems worldwide. Hospitals, in particular, face increasing pressure to balance rising demand with finite infrastructure and workforce capacity. Demographic changes, such as population aging, the growing prevalence of chronic diseases, and unexpected shocks like epidemics, place continuous strain on healthcare delivery. Within this context, the management of hospital beds emerges as a critical operational concern.

Effective bed management is closely linked to both cost efficiency and quality of care [1, 2]. Insufficient bed availability can trigger a chain of adverse outcomes, including prolonged patient waiting times, compromised care quality [3], and increased levels of stress and burnout among hospital staff [4]. These challenges are further compounded by the variability of patient demand, which is shaped by variations in disease incidence and the unpredictable onset of health crises.

Hospital bed demand and occupancy forecasting is a topic with multiple scales and perspectives. On the strategic level, optimizing national plans regarding hospital infrastructure involves a large spatial scale combined with a large temporal scale [5–7]. At the tactical level, medium-term horizons of several weeks to months are vital for workforce scheduling and vacation planning to ensure continuous service provision despite seasonal fluctuations in demand [8–11]. At the operational level, short-term predictions in the range of few hours, are particularly relevant for managing resources in emergency departments and intensive care units [12–17]. To address these challenges, accurate forecasting of hospital bed occupancy has become an increasingly important area of research [18].

Approaches to predict future bed occupancy can be broadly categorized into two main types: indirect methods and direct time series forecasting. Indirect methods involve forecasting key components that contribute to bed occupancy, such as predicting patient arrivals and the lengths of stay (LoS) of admitted patients, and then combining these forecasts to estimate future bed needs or occupancy levels [19–22]. This approach often requires detailed patient-level data and can involve complex modeling for LoS prediction. In contrast, direct time series forecasting leverages historical bed occupancy patterns to project future levels without the need for sensitive patient-specific information. The suitability of these approaches depends on the forecasting horizon. Indirect methods, particularly those based on patient flow dynamics, are often valuable for very short-term predictions. Direct time series forecasting is widely applied across medium-term and long-term horizons.

The existing literature on bed occupancy forecasting demonstrates the application of a variety of modeling approaches. Traditional statistical time series models, such as Autoregressive Integrated Moving Average (ARIMA) [23] and Seasonal ARIMA (SARIMA) [14], have been employed to capture temporal dependencies and seasonality in bed occupancy data. Regression based models and other machine learning techniques like Support Vector Regression (SVR) [24, 25] and ensemble methods such as Random Forest [24, 26, 27], and Gradient Boosting [24, 26] have also been explored to predict bed demand and occupancy. These models often incorporate various exogenous factors to improve forecasting accuracy and offer valuable insights into capturing various patterns in bed occupancy data.

Deep learning models have also been applied to the problem of hospital bed occupancy forecasting, particularly for their ability to model complex non-linear relationships and temporal dynamics. Recurrent Neural Networks (RNNs), particularly Long Short-Term Memory (LSTM) networks and Gated Recurrent Units (GRUs), have demonstrated significant potential in capturing complex temporal dependencies in bed occupancy [5, 10, 28, 29]. Transformer based models have also been used [30, 31].

While complex machine learning and deep learning models have demonstrated strong performance in various time series forecasting tasks, their complexity and the lack of sometimes interpretability at times hinder their practical implementation in a clinical setting. Prophet [32] is a generalized additive model specifically engineered to forecast time series exhibiting multiple seasonalities, trend changepoints, and holiday effects, all with minimal parameterization and full transparency of its decomposed components. Prophet has been applied to various healthcare forecasting tasks including patient admissions and discharges [33, 34], infectious disease trends [35, 36], and healthcare resource planning [37]. While the predictive performance of Prophet in these studies has been mixed when compared to other methods, Prophet remains attractive due to its interpretability, ease of use, and flexibility. Despite its relevance, Prophet’s role in mid-term hospital bed occupancy forecasting has not been systematically examined. This study addresses that gap by evaluating Prophet’s performance in this domain.

Furthermore, effective operationalization is crucial for ensuring that forecasts translate into actionable insights for hospital staff and administrators. While the primary focus of this work is on forecasting hospital bed occupancy, particular attention was also given to the operational integration of the forecasting outputs. To support practical decision-making, we implemented a production-grade data pipeline and role-specific dashboards, allowing forecasts to be regularly updated and made accessible to relevant stakeholders. Although such implementation details related to data infrastructure, processing, and delivery platforms are essential for enabling the actual use of forecasts in clinical and administrative settings, they are often omitted in related work. By sharing our approach to data infrastructure and delivery, we aim to contribute to the growing interest in making predictive models not only accurate but also actionable.

## Methods

### Data acquisition and preprocessing

We collected administrative data of inpatient stays from the Medical Center – University of Freiburg between January 1, 2010, and December 31, 2023. The dataset comprises only the following operational variables: the location identifier, the admission time, and the discharge time. No personally identifiable information was collected, processed or analyzed.

**Fig. 1:**
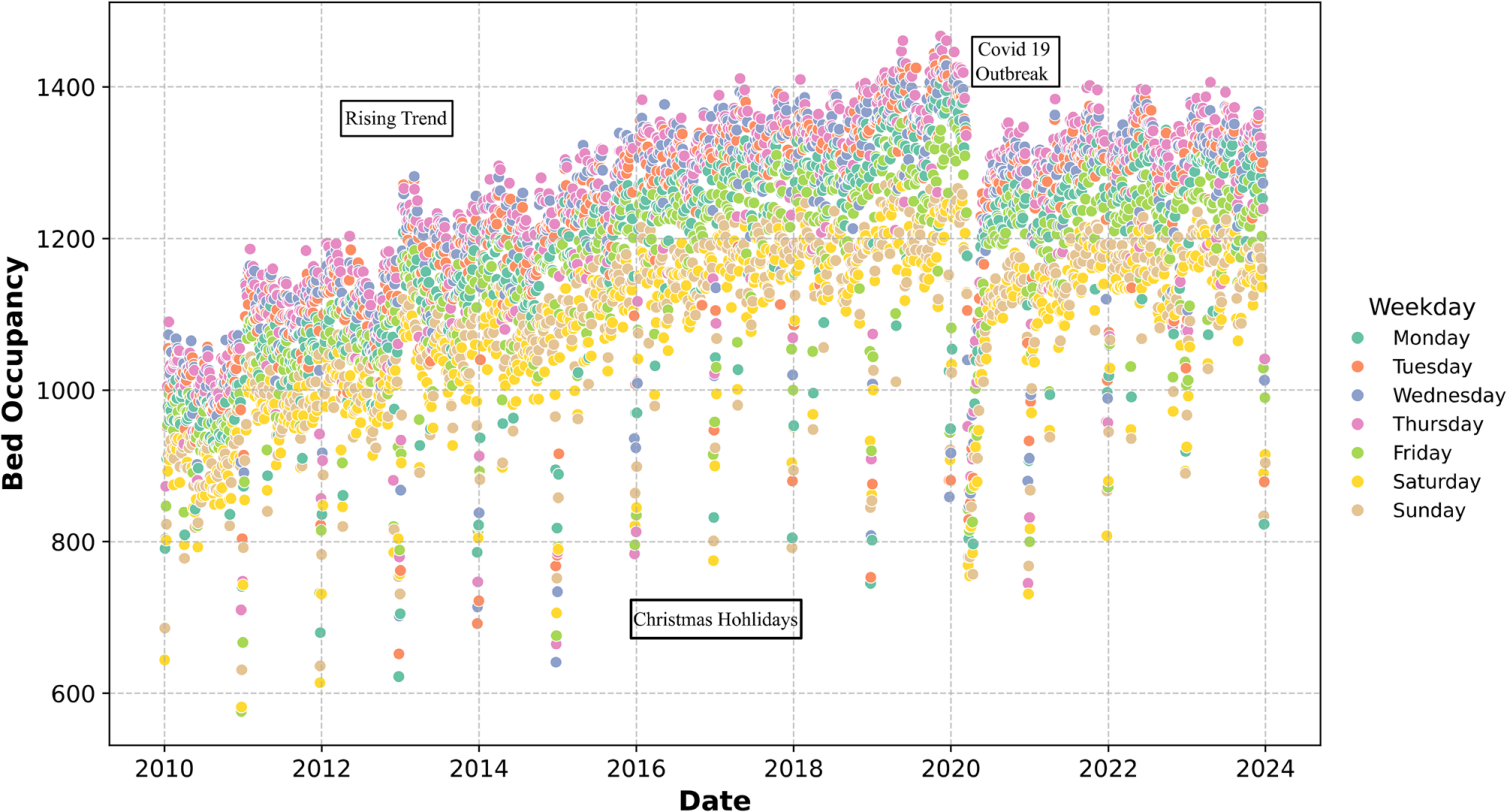
Daily hospital bed occupancy between January 2010 and December 2023 at the Medical Center – University of Freiburg. The scatter plot depicts the complete time series of daily bed occupancy, with points colored by weekday to highlight weekly seasonality. A long-term upward trend is visible, reflecting gradual capacity expansion and increasing service demand, temporarily interrupted by the decline during the first wave of the COVID-19 pandemic in 2020. In addition to this overall trajectory, regular weekly and yearly seasonal patterns are apparent, with occupancy peaking on weekdays and decreasing over weekends and major holidays such as Christmas, consistent with operational scheduling and reduced hospital activity during these periods

To transform these records into a time series format suitable for forecasting, we aggregated the daily admission and discharge counts to derive daily bed occupancy. We defined the daily bed occupancy as the number of occupied beds at midnight. The resulting time series illustrated in Fig. 1 represents the daily bed occupancy used in this study. Visual inspection reveals a clear upward trend over the years, which can be plausibly linked to the gradual expansion of hospital capacity and growing service demand. This trajectory is interrupted during the first wave of the COVID-19 pandemic in 2020, when occupancy temporarily declined, consistent with the suspension of elective care and reduced patient admissions during that period. Beyond these long-term developments, recurring seasonal structures are evident. At the weekly level, occupancy peaks on weekdays and declines over weekends, reflecting operational practices in which elective procedures are concentrated during the working week, whereas weekends are characterized by reduced activity and increased discharges due to limited staff availability. At the yearly level, marked reductions are observed around major holidays, most prominently at Christmas, when hospital activity is substantially curtailed. These descriptive insights informed the modelling strategy to include weakly and yearly seasonalities and motivated the inclusion of the following exogenous variables:

- **Public holidays**: A binary variable indicating the presence of public holidays, which often correlate with changes in hospital admissions and discharges. Given that the hospital is located in Baden-Württemberg, we used a list of national and regional public holidays relevant to this state.
- **COVID-19 pandemic period**: A special one-time holiday variable was created to account for the unique impact of the COVID-19 pandemic on hospital operations. This variable spanned the period from the 20th of March to the 30th of June 2020, effectively flagging the first pandemic’s lockdown duration as a distinct event with significant influence on bed occupancy.

### Model and forecasting methodology

To forecast daily hospital bed occupancy, we employed Prophet developed by Facebook (now Meta). Prophet is a generalized additive model (GAM), which decomposes the time series into trend, seasonality, and holiday components, offering a robust framework for modeling time series data with complex seasonal patterns. This structure is directly aligned with the patterns observed in Fig. 1, which makes Prophet particularly well-suited for mid-term hospital bed occupancy forecasting. Prophet can be expressed as:

$$\:\mathrm{y}\left(\mathrm{t}\right)\hspace{0.17em}=\hspace{0.17em}\mathrm{g}\left(\mathrm{t}\right)\hspace{0.17em}+\hspace{0.17em}\mathrm{s}\left(\mathrm{t}\right)\hspace{0.17em}+\hspace{0.17em}\mathrm{h}\left(\mathrm{t}\right)\:+\:\varepsilon \left(\mathrm{t}\right)$$

where:

- *y(t)* denotes the bed occupancy at time *t*.
- *g(t)* represents the trend function, which models non-periodic changes in bed occupancy. We utilized a piecewise linear trend, allowing for the accommodation of varying growth rates and the identification of trend change points.
- *s(t)* captures seasonal variations, including weekly and yearly patterns. These were modeled using a Fourier series, with the flexibility to adjust the number of terms to accommodate varying degrees of seasonal complexity. We set the number of Fourier components for the yearly and weekly seasonalities to 10 and 3, respectively.
- *h(t)* models the effects of holidays and special events, such as public holidays and the COVID-19 pandemic period. By incorporating these as regressor variables, we explicitly can account for their impact on bed occupancy.
- *ε(t)* is an error term, representing idiosyncratic variations not explained by the model components. The error term is modeled under the parametric assumption of normality.

To generates prediction intervals, Prophet simulates future trend changes based on historical rate variations at changepoints. The model infers the distribution of past rate changes to project potential future trend scenarios.

Preliminary hyperparameter tuning was conducted using a separate validation set to assess the impact of key Prophet parameters, such as changepoint flexibility and seasonal Fourier terms. However, these experiments revealed no substantial improvements over the default settings^1^. Consequently, default hyperparameters were retained to maintain model simplicity without sacrificing accuracy.

### Evaluation

To evaluate the forecasting performance, we conducted a rolling cross-validation for the two-year period from January 1, 2022, to December 31, 2023. This involved training the model on a subset of the data, making predictions for the next 30, 60, 90, and 180 days, and comparing these predictions to the actual observed values.

Specifically, we employed a rolling-origin evaluation approach. For every calendar month in evaluation period, the model was trained from scratch on the training data from January 1, 2010, up to the beginning of that month. Subsequently, forecasts were generated for the following 30, 60, 90, and 180 days. To ensure the test split for each fold fully covered the forecast horizon, we varied the final cutoff date of the evaluation set by horizon, resulting in 24 folds for 30 days, 23 folds for 60 days, 22 folds for 90 days, and 19 folds for 180 days. With this setup, the training set size for a given fold was identical across horizons extending the period from 2010 up to the cutoff date, while the test set size was determined by the respective forecast horizon. Because the cutoffs were spaced at monthly intervals, this design introduced partial overlap across folds. For the 30-day horizon, overlap was minimal, whereas for the 60- and 90-day horizons, many dates were forecast two or three times and up to 6 times for the 180-day horizon. This overlap is a natural property of rolling-origin evaluation when fixed forecast horizons extend beyond the fold spacing. While this overlap induces correlation among performance metrics across folds, it does not bias the estimation of the performance metrics of the model.

We used the following metrics to assess the accuracy of our forecasts:

- The **Root Mean Squared Error (RMSE)** provides an interpretable measure of the standard deviation of these errors.
- The **Mean Absolute Error (MAE)** calculates the average magnitude of errors, giving a direct sense of the error’s scale in the original units of measurement.
- The **Mean Absolute Percentage Error (MAPE)** expresses prediction accuracy as a percentage, specifically as the average absolute percentage difference between predicted and actual values.
- The **Median Absolute Percentage Error (MDAPE)** offers a perspective on the median of the absolute percentage errors, which can be particularly useful in datasets with skewed error distributions.
- The **Symmetric Mean Absolute Percentage Error (SMAPE)** is a variant of MAPE that ensures equal weighting for over and under-prediction errors, providing a balanced view of prediction accuracy.
- Lastly, **Coverage** assesses the model’s reliability by calculating the percentage of actual values that fall within the model’s predicted confidence intervals.

While Prophet formally assumes normally distributed residuals, in the context of daily hospital bed occupancy, the error term may not follow an exact Gaussian distribution, as fluctuations and occasional shocks (e.g., atypical admission increases or sudden pandemic outbreaks) can generate asymmetric or heavy-tailed errors. To obtain statistically robust estimates of the model performance, we applied a non-parametric bootstrapping at the level of individual forecast–observation pairs generated during cross-validation. These pairs were resampled with replacement, and performance metrics were recomputed for each bootstrapped sample. A total of 10,000 bootstrap samples were generated, with the 2.5th and 97.5th percentiles of the resulting distributions taken as the bounds of the 95% confidence intervals. By resampling directly from the empirical distribution of forecast errors, the bootstrapping provides both consistent point estimates and empirical quantification of uncertainty across all reported metrics. This approach mitigates the impact of non-Gaussian residual behavior by providing distribution-free uncertainty quantification.

To ensure transparency and reproducibility, the complete set of scripts used to generate the results and figures reported in this study is publicly accessible via a dedicated GitHub repository at https://github.com/mcuf-idim/bed-occupancy-forecasting-with-prophet.

### Data pipeline and dashboard implementation

**Fig. 2:**
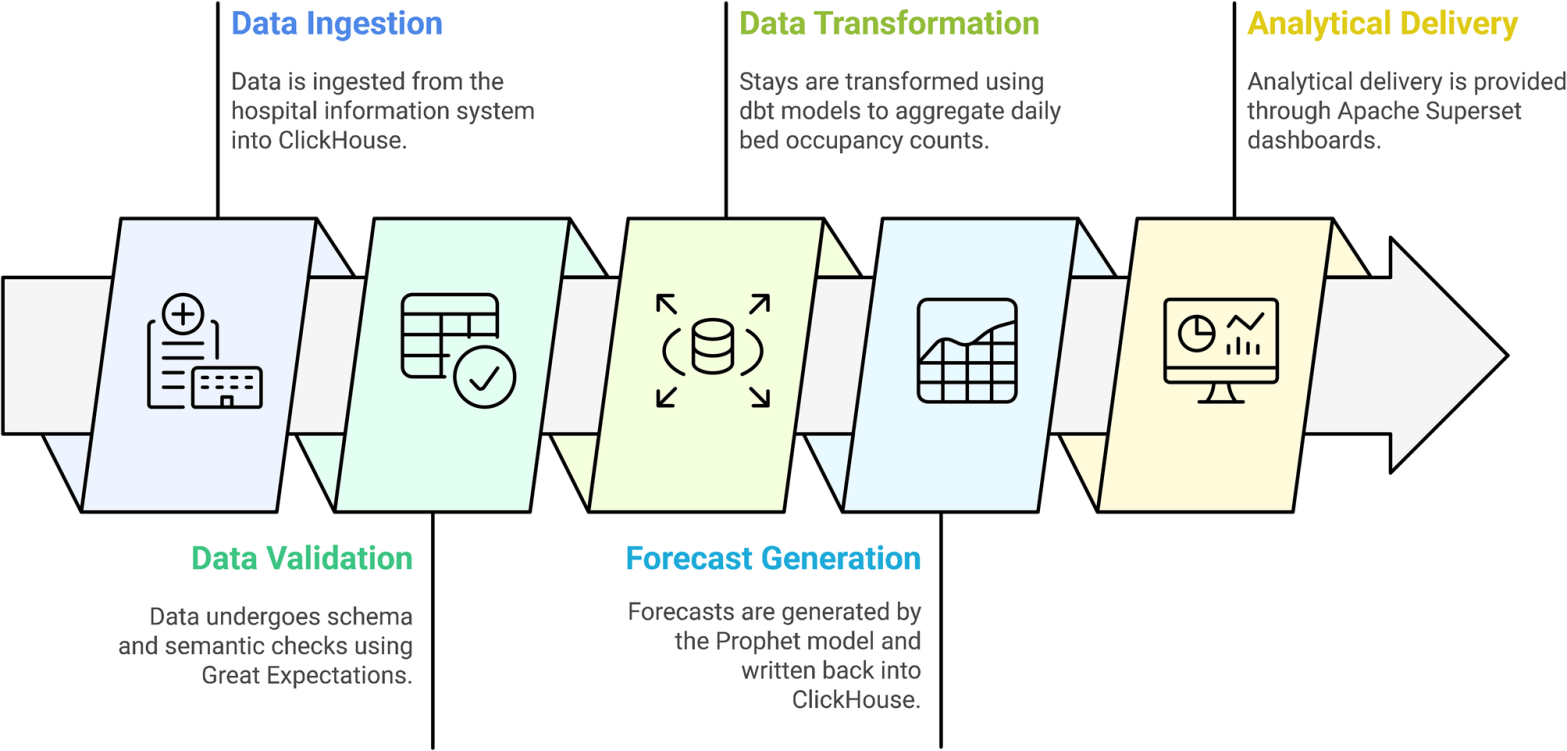
High-level workflow diagram for hospital bed occupancy forecasts. This diagram illustrates the key stages of the data pipeline, including data ingestion from the hospital information system into clickhouse, data validation using great expectations, data transformation via dbt models, forecast generation using the prophet model, and analytical delivery through apache superset dashboards

To ensure end-to-end reproducibility and operational reliability, we developed data pipelines based on cloud-native technologies that orchestrates ingestion, validation, transformation, storage and delivery of both raw admission data and forecast outputs. A high-level workflow diagram is illustrated in (Fig. 2). Stays records are ingested from the hospital information system into ClickHouse [38], a columnar OLAP database optimized for high-concurrency analytical queries. Following ingestion, raw data undergo schema and semantic checks using Great Expectations^2^; any violations trigger automatic alerts and halt downstream execution until anomalies are resolved. Subsequently, dbt models^3^ execute SQL-based transformations to aggregate cleaned records into daily bed occupancy counts.

Forecasts produced by the Prophet model are written back to ClickHouse and stored alongside historical occupancy data. Consolidating observed and forecasted data within a single analytical store simplifies downstream access and ensures consistency across dashboards and analytical outputs. Metadata schemas capture model versioning, execution timestamps, and performance metrics using MLFlow^4^, thereby supporting reproducibility and facilitating comparative evaluation across forecast iterations. Analytical delivery is implemented through Apache Superset^5^ dashboards, which connect directly to the ClickHouse data store.

**Fig. 3:**
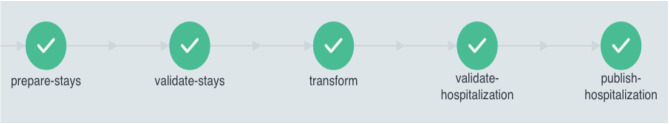
Argo-Workflow for the hospital bed occupancy data pipeline following the write-audit-publish pattern generated by Argo Web-UI

The pipelines are orchestrated by Argo Workflows^6^ on a Kubernetes cluster. Each task includes retry mechanisms with exponential backoff and employs immutable artifact storage to ensure that transient failures do not compromise data integrity or audit trails. All transformation steps are version-controlled and automatically documented, with lineage graphs generated to support traceability. The overall workflow adheres to the write-audit-publish data engineering pattern, as illustrated in Fig. 3.

## Results

### Model component analysis

**Fig. 4:**
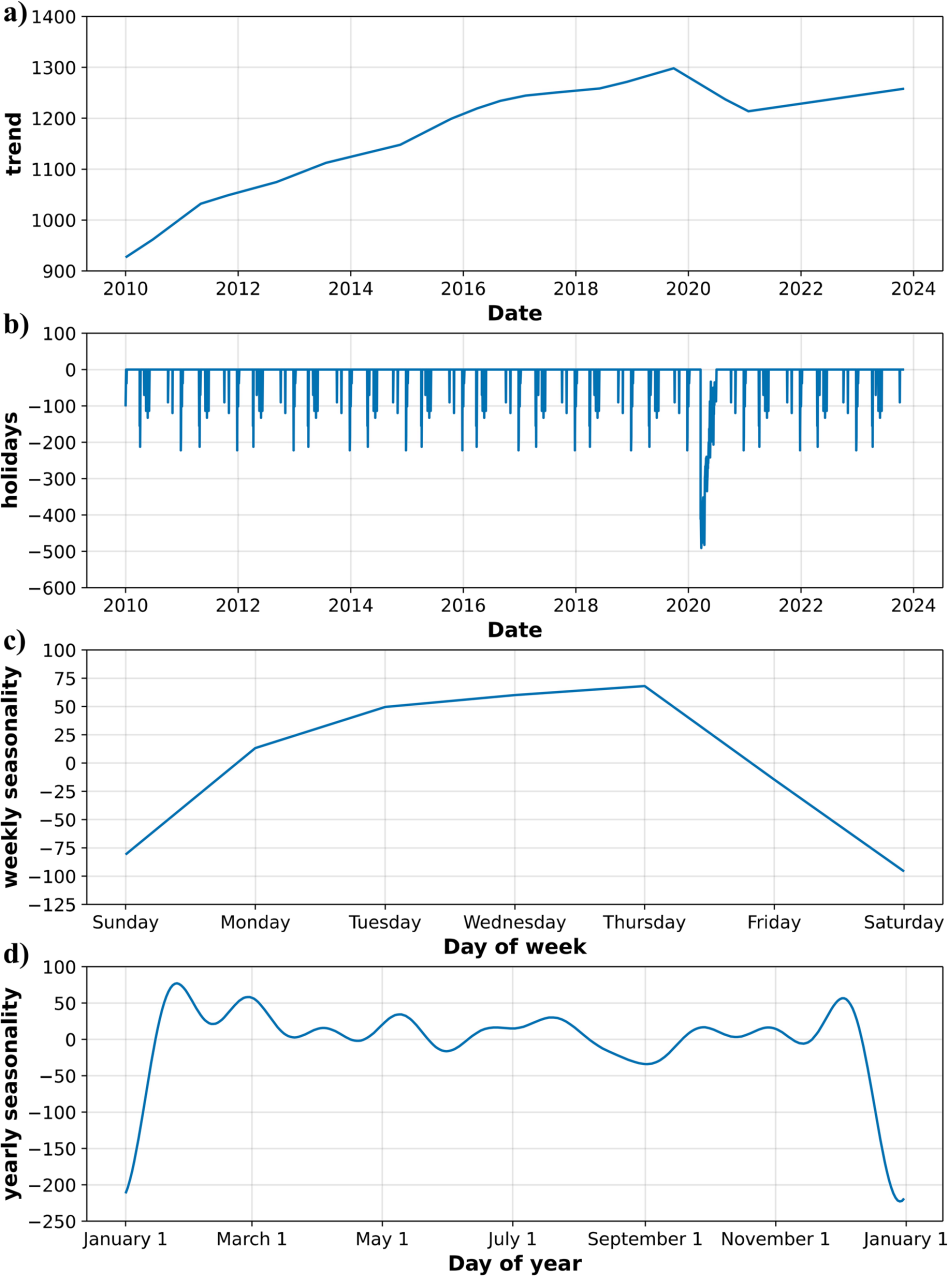
Decomposition of Prophet model components after training. (**a**) Long-term trend component showing a gradual increase in occupancy, with a decline during the COVID-19 pandemic. (**b**) Impact of public holidays and the early pandemic period, with consistent negative effects on occupancy. (**c**) Weekly seasonality revealing higher occupancy on weekdays and lower values on weekends. (**d**) Yearly seasonality capturing recurring patterns, including a pronounced dip around the end-of-year holidays. The components provide interpretable insights into temporal dynamics relevant for hospital capacity planning

The decomposition of the Prophet model after fitting the training data into its constituent components provides valuable insights into the underlying patterns driving hospital bed occupancy. As depicted in Fig. 4, the model reveals distinct trends, holiday effects, weekly seasonality, and yearly seasonality.

The trend component reflects the long-term growth observed in the raw data, including the sharp decline during the COVID-19 outbreak and the subsequent recovery. The holiday component captures the impact of specific events on bed occupancy. As expected, public holidays result in a noticeable decrease in occupancy, confirming the descriptive observation of reduced activity during these periods. The most significant negative impact is observed during the COVID-19 pandemic period. This highlights the substantial disruption caused by the pandemic, with a more pronounced effect than regular public holidays.

The weekly seasonality component reproduces the pronounced weekday–weekend cycle, capturing the operational rhythm of higher occupancy during weekdays and lower levels during weekends. The yearly seasonality component illustrates fluctuations in bed occupancy throughout the year. The observed pattern shows a sharp drop toward the end of the year could be attributed to the Christmas holiday period. A smooth cyclic structure of the yearly seasonality component can be noted, which reflects the Fourier series representation to model this component.

Taken together, these components are consistent with the descriptive patterns identified in the time series. The components provide a comprehensive understanding of the factors influencing hospital bed occupancy. The trend captures long-term changes and the impact of significant events like the COVID-19 pandemic. The holiday component quantifies the effects of specific events, while the weekly and yearly seasonality components highlight recurring patterns related to hospital operations and patient behavior. This detailed understanding can inform strategic planning and resource allocation, ensuring efficient hospital operations and patient care.

### Bed occupancy forecasting

To assess the accuracy and reliability of mid-term hospital bed occupancy forecasting, we evaluated Prophet using rolling cross-validation over multiple forecast horizons. The analysis focused on four planning intervals; 30, 60, 90, and 180 days, which are chosen to reflect common operational and tactical decision-making needs in hospital management. The corresponding forecasting performance is summarized in Table 1. The model’s accuracy was consistently high across horizons. Root Mean Squared Error (RMSE) estimates ranged from 53.46 (95% CI: 49.28–57.83) to 57.24 (95% CI: 55.44–59.04). Mean Absolute Error (MAE), which expresses the average magnitude of deviations in the same units as the data, ranged from 38.77 (95% CI: 36.17–41.51) to 43.07 (95% CI: 41.81–44.35), against an average of 1,230 daily occupied beds during the evaluation period (2022–2024). Mean Absolute Percentage Error (MAPE) varied between 3.21% (95% CI: 2.98–3.46) and 3.53% (95% CI: 3.42–3.64), while Median Absolute Percentage Error (MDAPE) was slightly lower, ranging from 2.37% (95% CI: 2.12–2.64) to 2.71% (95% CI: 2.58–2.84). Symmetric MAPE (SMAPE) produced comparable results, ranging from 3.20% (95% CI: 2.97–3.43) to 3.54% (95% CI: 3.43–3.65). Finally, coverage rates, reflecting the proportion of actual values lying within the prediction intervals, remained consistently above 80%, ranging from 80.37% (95% CI: 79.04–81.70) to 84.30% (95% CI: 81.53–86.94).

**Fig. 5:**
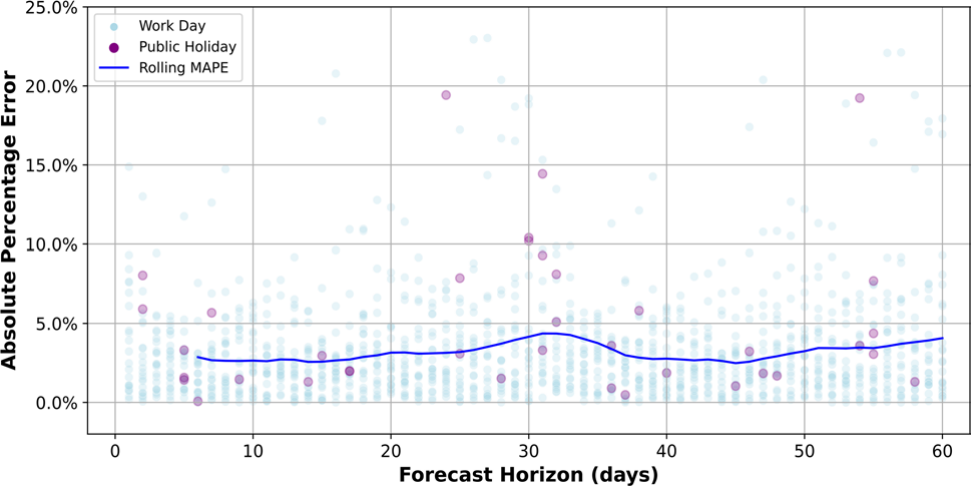
Mean absolute percentage error (MAPE) performance during rolling cross-validation. The figure displays a scatter plot of individual Absolute Percentage Error (MAPE) values for forecasts with a 60-day horizon derived from the cross-validation process, superimposed with the rolling mean (MAPE) to show the overall trend in prediction accuracy over the forecast horizon

While our bootstrap resampling procedure provides uncertainty estimates for aggregate performance metrics, it does not reveal how forecasting accuracy evolves across different points of the prediction horizon. To complement the aggregated evaluation, we illustrate horizon-specific variability in Fig. 5 which presents a detailed visualization of the Mean Absolute Percentage Error (MAPE) derived from the cross-validation process with a 60-day horizon. The scatter plot displays the MAPE for each individual prediction ranked by their forecast horizon (number of days ahead) so that points to the right correspond to predictions made further into the future. This ordering allows visual inspection of how error behaves as the horizon increases. Public holidays and normal work days indicated for reference. Superimposed on the scatter plot is the rolling mean of the MAPE, which smooths out the variability of individual values and reveals the overall trend in predictive accuracy. While the rolling mean highlights the consistent performance of the model, with MAPE values remaining below 5%, closer inspection reveals fluctuations in error magnitude. These fluctuations do not exhibit a systematic correspondence with holidays, suggesting that holiday effects were appropriately captured by the model. However, the error fluctuations appear to have a cyclic pattern every 30 days. This cyclic nature may point to limitations of the Fourier series approximation of yearly seasonality, which can introduce periodic error patterns.

**Fig. 6:**
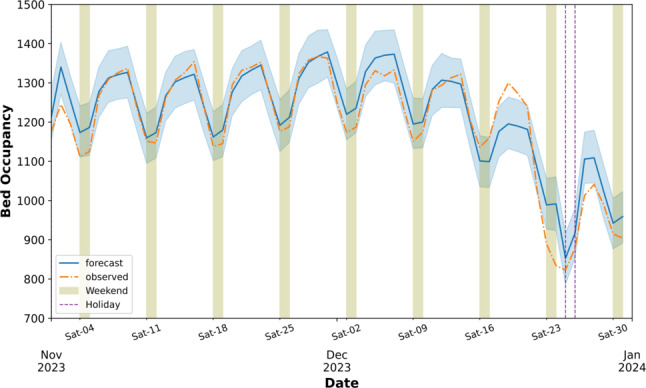
Observed versus 60-day forecasted hospital bed occupancy. This figure visually compares the actual observed daily bed occupancy values with the model’s 60-day forecast, illustrating the model’s ability to track observed fluctuations, including weekly patterns and the dip around the Christmas holiday period, highlighted by vertical dashed lines. The model was trained on data from January 2010 up to November 2023

Figure 6 depicts the 60-day forecast of bed occupancy alongside the observed values for the last cross-validation fold, providing a visual assessment of the model’s predictive accuracy. Notably, the model closely tracks the observed fluctuations in bed occupancy, capturing both the daily and weekly patterns. Furthermore, the model accurately predicts the dip in occupancy around the Christmas holiday period, highlighted by the vertical dashed lines.

**Table 1:** Bootstrapped performance metrics of Prophet across different forecast horizons. The table reports the estimated performance statistics and their corresponding 95% confidence intervals (CI). The results reflect consistently low error magnitudes and stable uncertainty estimates across all horizons, indicating robust forecasting

	30 days	60 days	90 days	180 days
RMSE (CI 95%)	53.46 (49.28–57.83)	53.70 (50.67–56.72)	54.76 (52.31–57.28)	57.24 (55.44–59.04)
MAE (CI 95%)	38.77 (36.17–41.51)	53.70 (50.67–56.72)	40.32 (38.72–41.96)	43.07 (41.81–44.35)
MAPE (CI 95%)	3.21% (2.98%-3.46%)	3.23% (3.06%-3.40%)	3.32% (3.18%-3.46%)	3.53% (3.42%-3.64%)
MdAPE (CI 95%)	2.37% (2.12%-2.64%)	2.38% (2.19%-2.54%)	2.47% (2.35%-2.62%)	2.71% (2.58%-2.84%)
sMAPE (CI 95%)	3.2% (2.97%-3.43%)	3.22% (3.05%-3.38%)	3.31% (3.17%-3.45%)	3.54% (3.43%-3.65%)
Coverage (CI 95%)	84.3% (81.53%-86.94%)	84.12% (82.17%-86.09%)	83.18% (81.57%-84.85%)	80.37% (79.04%-81.70%)

### Decision support via forecast dashboard integration

**Fig. 7:**
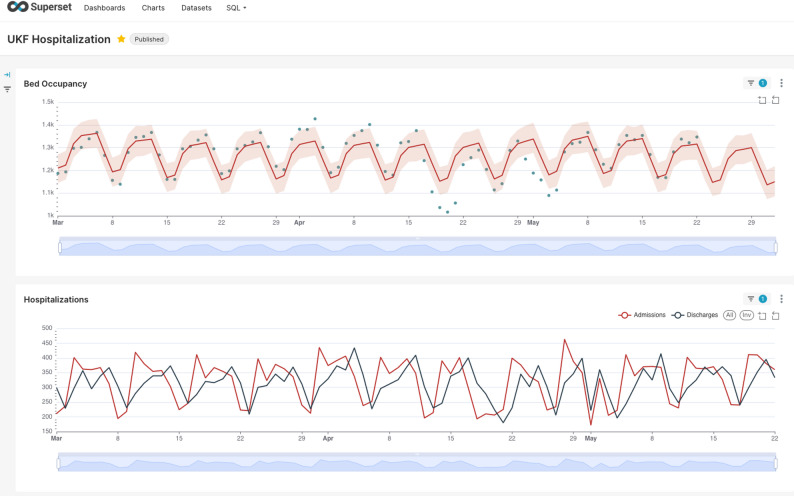
Superset dashboard visualization of hospital bed occupancy and hospitalization flows at the Medical Center – University of Freiburg from March to June 2025. (Top) Forecasted daily bed occupancy (red line) with prediction intervals (shaded area) and observed values (dots). (Bottom) Daily admission and discharge volumes, illustrating operational fluctuations and weekly seasonality

To facilitate the operational use and interpretability of the forecasting results, we implemented an interactive dashboard that visualizes both historical and forecasted bed occupancy (Fig. 7). The first panel presents a time series plot comparing observed occupancy levels with Prophet-generated forecasts, enabling stakeholders to visually assess model accuracy and anticipated trends. The second panel displays daily admission and discharge counts, providing contextual insight into occupancy dynamics. These visualizations, integrated into the analytics workflow, support real-time monitoring and retrospective validation, and are intended to assist hospital administrators in short- to mid-term capacity planning.

## Discussion

This study demonstrates the effectiveness of the Prophet model in forecasting hospital bed occupancy. By incorporating public holidays and the COVID-19 pandemic, the model was able to capture the complex dynamics underlying bed occupancy. The component analysis revealed interpretable patterns that align closely with domain knowledge and operational realities in hospital management. The trend component reflected the expansion of hospital services up to the onset of the COVID-19 pandemic, followed by a marked decline and gradual recovery, mirroring the well-documented disruptions in hospital operations during this period. Weekly seasonality captured higher occupancy during weekdays and reduced levels on weekends, consistent with elective procedure scheduling and discharge practices. The model also successfully isolated the effects of public holidays and special events, such as the sharp decline in admissions during Christmas and the compounded disruptions caused by pandemic-related constraints.

The performance evaluation across different forecasting horizons remained robust, with mean absolute percentage errors (MAPE) consistently below 4%. The low dispersion between metrics such as MAPE and SMAPE together with narrow confidence intervals, indicates that forecast errors were not only small on average but also stable across different horizons. Similarly, the relatively small gap between mean and median absolute percentage errors suggests robustness to outliers, underscoring the model’s resilience across varying occupancy scenarios. Furthermore, coverage values between 80% and 84%, with overlapping confidence intervals, underscore the reliability of the predictive intervals. Operationally, this indicates that approximately four out of five future occupancy values fell within the model’s uncertainty bounds, a practically useful benchmark for medium-term planning. While not reflecting perfect calibration, this level of coverage provides decision-makers with credible ranges to support resource allocation, staffing, and contingency planning under realistic uncertainty.

Several studies suggest that relatively simple time‑series forecasting models can deliver reliable forecasts of inpatient bed occupancy. For example, Earnest et al. [39] applied a seasonal ARIMA (1,0,3) model to predict isolation‑bed usage during Singapore’s 2003 SARS outbreak and achieved three‑day forecast MAPE of 8.6%. At a national scale, Shah et al. [9] evaluated exponential smoothing, SARIMA and TBATS across 121 NHS trusts and reported median percentage errors ranging from 2.45% in summer to 4.91% in winter, all without recourse to neural networks. Vollmer et al. [26] similarly found that penalized linear models outperformed a range of machine‑learning and deep‑learning methods for short‑ to medium‑term forecasts across multiple hospital sites. Consistent with these findings, our Prophet‑based approach achieved MAPEs of 3.21% and 3.23% for 30‑ and 60‑day horizons, markedly improving upon the 5.55% and 5.48% reported by Kutafina et al. [10].

Although the literature presents mixed results regarding Prophet’s predictive performance in healthcare and hospital management domains, these inconsistencies largely reflect the diversity of forecasting tasks and horizons within these domains. Forecasting accuracy is highly contingent upon the specific target variable, the temporal resolution, and the forecast horizon; be it emergency department (ED) arrivals [33, 40, 41], discharge volumes [34, 42], or inpatient bed occupancy [37, 40]. Nevertheless, evidence from the literature reinforces the notion that model simplicity often suffices, even under high volatility. For example, Tuominen et al. [30] found that LightGBM outperformed deep-learning approaches for hourly ED census forecasting, while Caldas et al. [31] reported only marginal gains from a Temporal Fusion Transformer compared with simpler models. Such findings suggest that additive or tree-based approaches, which readily capture seasonality and holiday effects, can match or surpass more complex methods while remaining easier to interpret, automate, and maintain.

In the context of mid-term hospital bed occupancy forecasting, the results of this study demonstrate the effectiveness of the Prophet model, reflected in its notably low MAPE values. These findings raise the question of whether the added complexity of advanced forecasting architectures is warranted for this specific task and time horizon. Unlike highly volatile targets such as ED arrivals, bed occupancy is constrained by physical capacity, which inherently limits its variability [43]. In stable operational conditions, it is further governed by predictable patterns such as weekly cycles and seasonal trends [44]. Models based on decomposable time series analysis, like Prophet, are expressly designed to capture such structured components [35]. Although Tuominen et al. [40] argue that bed occupancy is more difficult to predict than arrivals due to variability in length of stay (LoS), their findings pertain primarily to very short-term horizons (e.g., one-day-ahead forecasts), where the stochastic nature of individual patient flows plays a greater role. For mid-term forecasting, where such fluctuations tend to smooth out, Prophet’s ability to model aggregate trends and seasonality becomes more advantageous.

Research consistently demonstrates that increasing model complexity often results in diminishing returns, with only marginal gains in predictive accuracy accompanied by significantly higher computational costs and maintenance burdens. This pattern appears across various domains, where marginal accuracy improvements rarely justify significant increases in computational resources and maintenance requirements [45, 46]. Prophet offers several practical benefits in this regard, most notably its minimal need for hyperparameter adjustment. Reducing reliance on extensive optimization is operationally advantageous, as it reduces computational costs and maintenance burdens associated with repeated fine-tuning, while enabling rapid deployment in settings with limited machine learning expertise. By minimizing the need for extensive optimization, efforts can instead focus on integrating forecasts into clinical decision-making.

While Prophet demonstrated promising results, our study has several limitations. The model’s accuracy may be influenced by the availability and quality of historical data. Additionally, it assumes that past patterns will persist into the future, an assumption that may not hold in the face of sudden systemic changes. Although the mean and median absolute percentage error values were close to 5%, occasional outliers could limit the model’s practical utility for high-stakes decision-making in hospital operations. The cyclic nature of the outliers might reflect limitations of the Fourier series representation of yearly seasonality. These fluctuations warrant further investigation and could be addressed in future work. Furthermore, the representation of the COVID-19 pandemic was simplified to a binary indicator, capturing its overall disruptive effect but not its multi-phase dynamics or epidemiological drivers such as infection and hospitalization rates. While this abstraction was sufficient for the present analysis, richer epidemiological data could help disentangle pandemic-specific effects in future studies.

Beyond model accuracy, this work presents the integration of forecasting models into a production-ready, end-to-end system architecture. The implementation of a cloud-native, modular data pipeline supported the generation of reliable, high-frequency forecasts of hospital bed occupancy. Forecast results were made accessible via analytical dashboards, providing clinical and administrative staff with interactive views of both historical and forecasted occupancy, to bridge the gap between predictive output and day-to-day decision-making.

## Conclusions

Accurate forecasting of hospital bed occupancy is essential for optimizing resource allocation and ensuring efficient patient care. This study demonstrates that Prophet offers a robust, interpretable, and operationally viable solution for mid-term hospital bed occupancy forecasting. Despite the availability of more complex forecasting methods, our results show that Prophet achieves competitive predictive accuracy, while providing the added benefits of simplicity, transparency, and ease of integration into clinical workflows. The model effectively captures key temporal dynamics such as weekly seasonality, holiday effects, and long-term trends, offering valuable insights for capacity planning and resource allocation. These findings support the broader argument that, for structured and relatively stable healthcare forecasting tasks, simpler models may outperform more complex alternatives not only in accuracy, but also in reproducibility, scalability, and usability.

## Supplementary Information

Below is the link to the electronic supplementary material.

Supplementary Material 1

## Data Availability

The datasets analyzed during the current study are not publicly available due to the hospital regulations, but are available from the corresponding author on reasonable request.

## Footnotes

1 A comprehensive description of the hyperparameter optimization study can be found in the Supplementary Materials.

2 https://github.com/great-expectations/great_expectations.

3 https://github.com/dbt-labs/dbt-core.

4 https://github.com/mlflow/mlflow.

5 https://github.com/apache/superset.

6 https://github.com/argoproj/argo-workflows.
