# Supplementary material for: Forecasting hospital bed occupancy: a time series approach with prophet
Source: BMC Med Inform Decis Mak. 2026 May 8;26:162. doi: 10.1186/s12911-026-03542-w (PMC13156892; doi:10.1186/s12911-026-03542-w)

**Hyperparameter Optimization**

To validate the robustness of the default parameter settings and ensure that utilizing a simple model did not compromise accuracy, a systematic hyperparameter optimization (HPO) study was conducted for the Prophet model.

### **Methodology**

The optimization process utilized the **Optuna** framework, which employed the **Tree-structured Parzen Estimator (TPE) sampler** to efficiently explore the parameter space. The optimization objective was to **minimize the Root Mean Squared Error (RMSE)**. The models were evaluated using cutoffs generated between **January 1, 2018** and **January 1, 2020**.

1. **Number of Trials:** The optimization was performed over **100 trials (NUM_TRIALS = 100)** for each forecast horizon. The initial **10 trials (NUM_STARTUP_TRIALS = 10)** were reserved for initial exploration to warm up the TPE sampler.

### **Tuned Parameters and Search Space**

We focused on tuning the three primary prior scales in the Prophet model, as these govern the complexity and flexibility of the component fits.

| **Parameter** | **Search Space**  **(Log Scale)** |
| --- | --- |
| changepoint_prior_scale | 0.001 to 0.1 |
| seasonality_prior_scale | 0.01 to 10 |
| holidays_prior_scale | 0.01 to 10 |

**Fixed Parameters**

The following parameters were held constant during the HPO study:

- **Seasonality Mode:** Set to **"additive"**.
- **Exogenous Variables:** the public holidays and the COVID-19 pandemic indicator, were included as a fixed input.
- **Fourier Terms:** The number of Fourier components for yearly (10) and weekly (3) seasonality were kept at the selected values defined in the methodology.

### **Results**

The optimization study was conducted for forecast horizons of 30, 60, 90, and 180 days. **Figure X** illustrates the history of the objective value (RMSE) across 100 trials for each horizon, comparing the default hyperparameter setting (yellow star) against the best optimized parameters (green cross). The objective value remains relatively stable across all trials, and the RMSE achieved by the default parameters is highly competitive with—or nearly identical to—the best RMSE found after 100 optimization trials. The comparison of RMSE values between the default settings and the best optimized settings is summarized below, demonstrating marginal gains achieved by the optimization:

| **Horizon** | **Default HPs RMSE** | **Best HPs RMSE** | **Optimization Improvement (Absolute RMSE)** |
| --- | --- | --- | --- |
| **30 Days** | 57.04 | 56.84 | 0.20 |
| **60 Days** | 56.31 | 56.14 | 0.17 |
| **90 Days** | 55.63 | 55.49 | 0.14 |
| **180 Days** | 55.47 | 55.26 | 0.21 |

**Forecasts plots for all horizons**


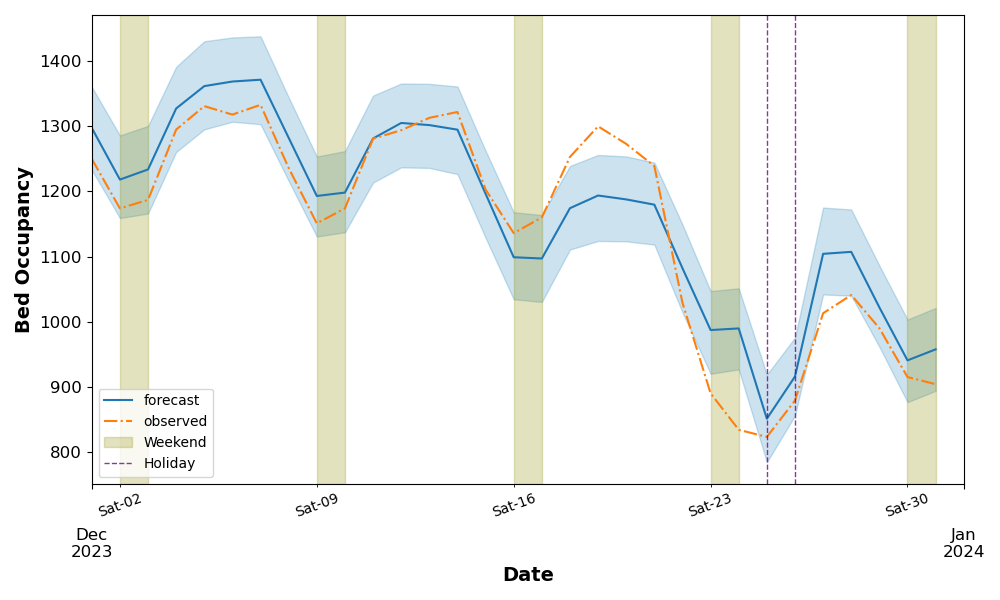

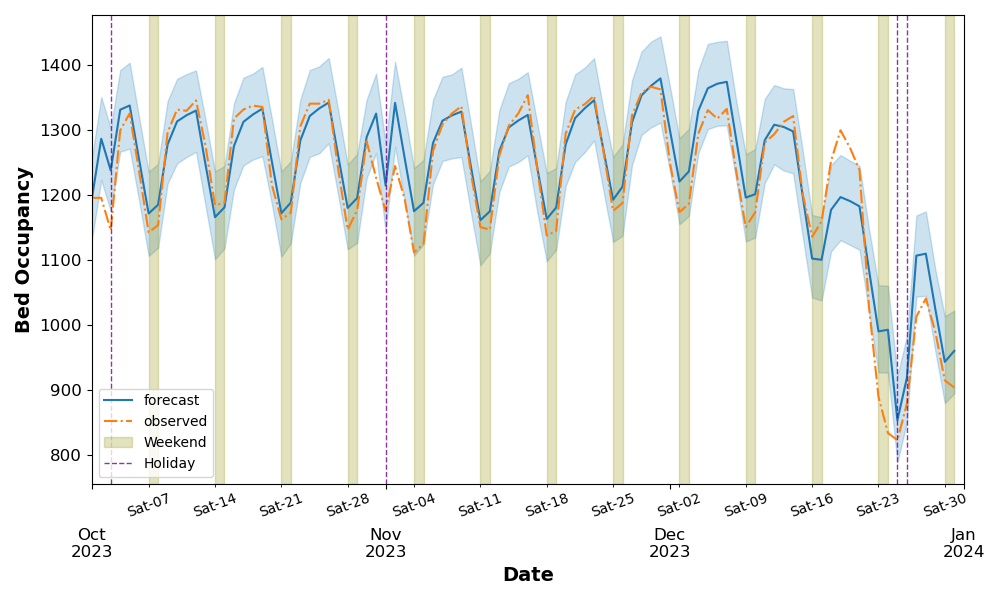

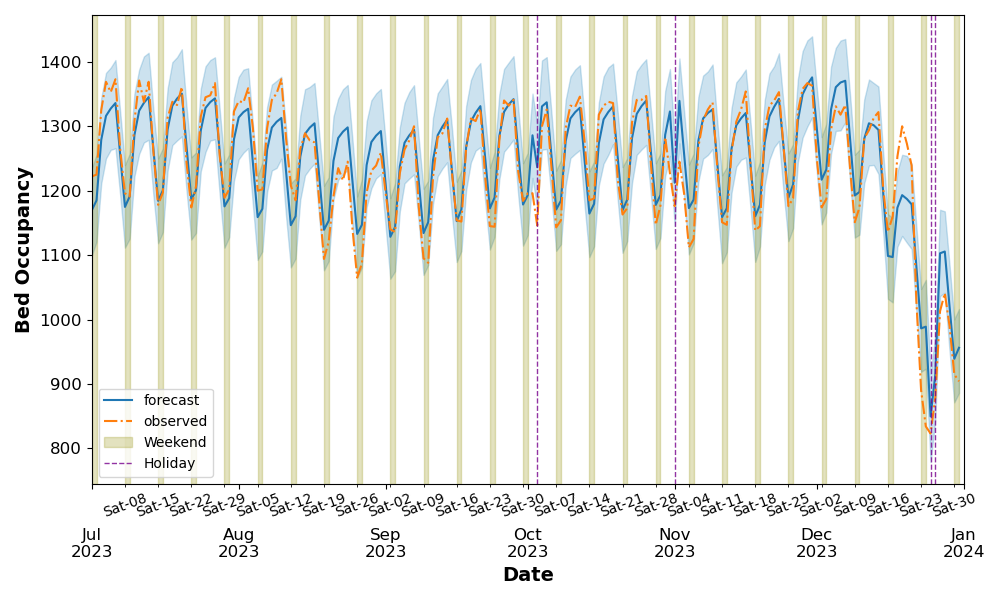


#
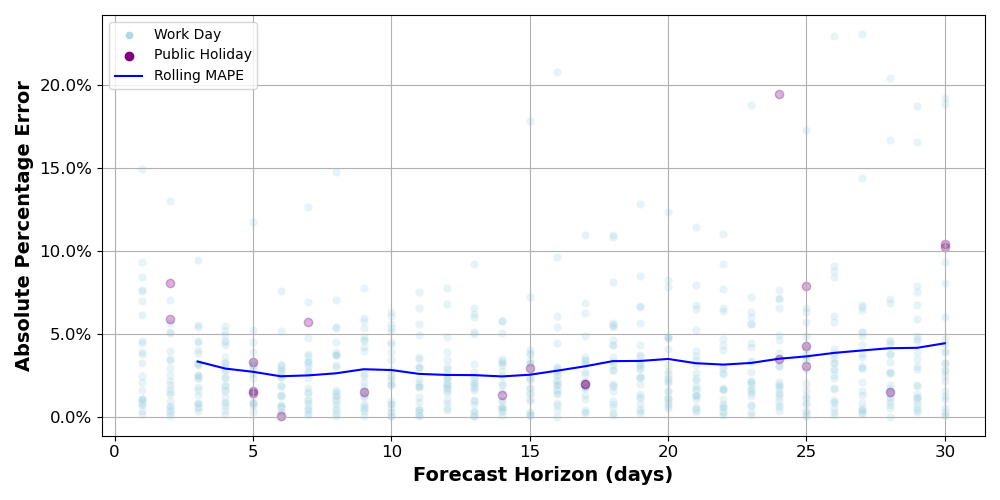

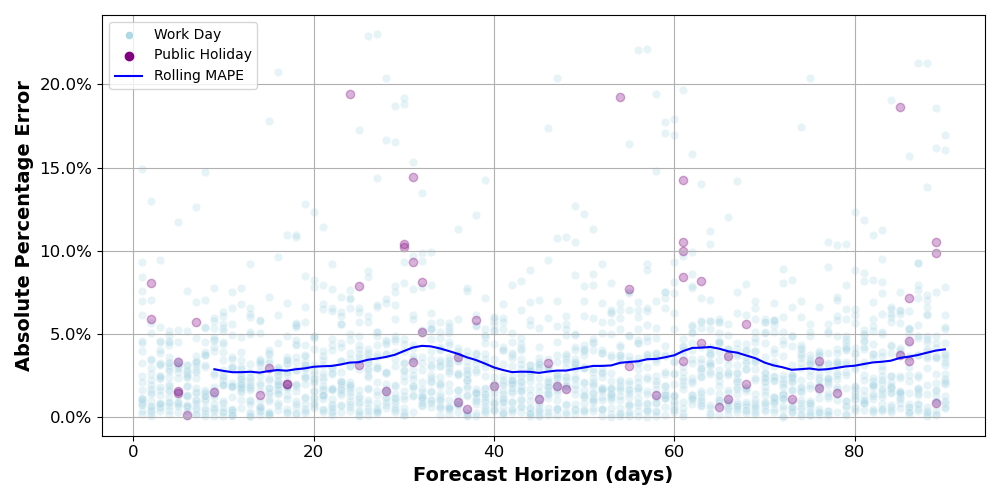

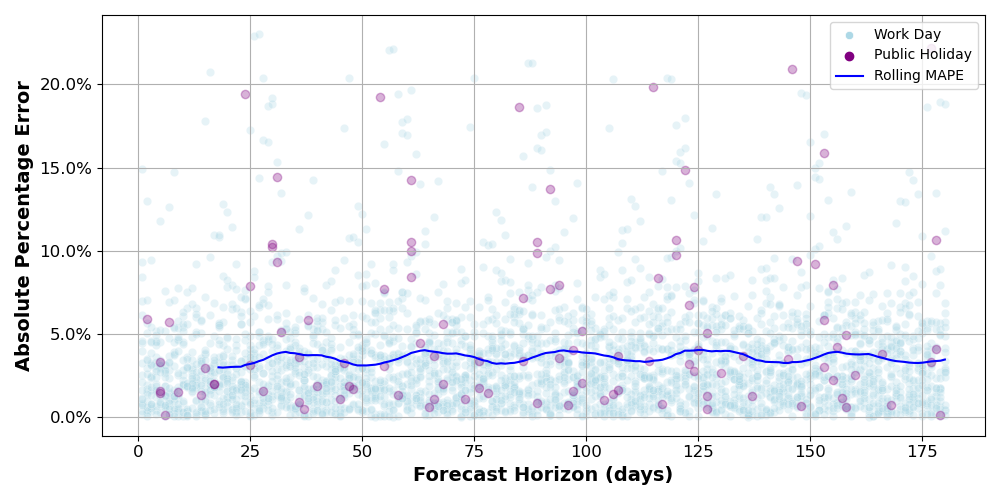

Supplement: Supplementary file 1 — Supplementary Material 1 [file 12911_2026_3542_MOESM1_ESM.docx]
